# Supplementary figures and images for: LINC00844 promotes proliferation and migration of hepatocellular carcinoma by regulating NDRG1 expression
Source: PeerJ. 2020 Jan 28;8:e8394. doi: 10.7717/peerj.8394 (PMC6993750; doi:10.7717/peerj.8394)

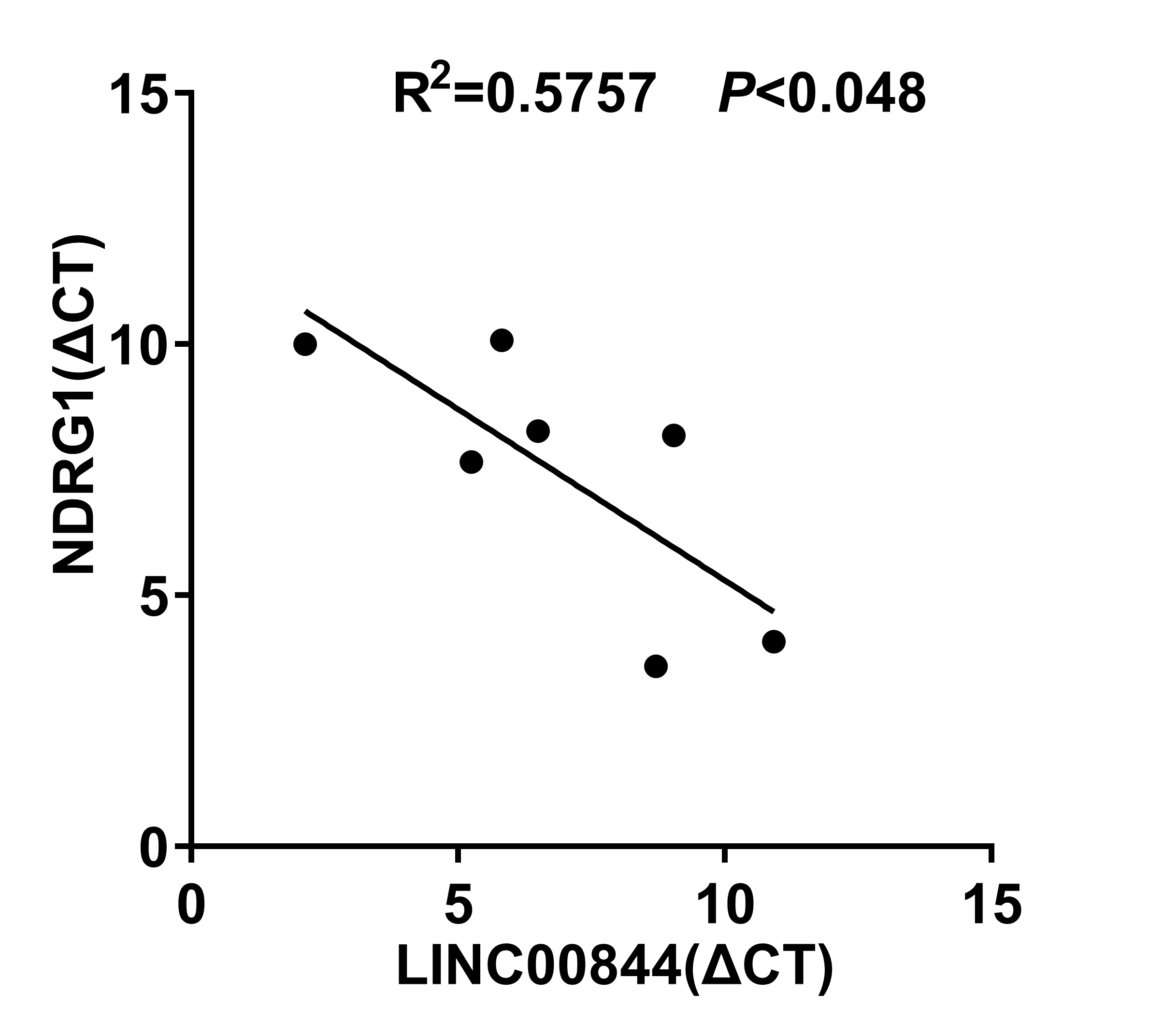

Supplement: Figure S1 [file peerj-08-8394-s001.png]

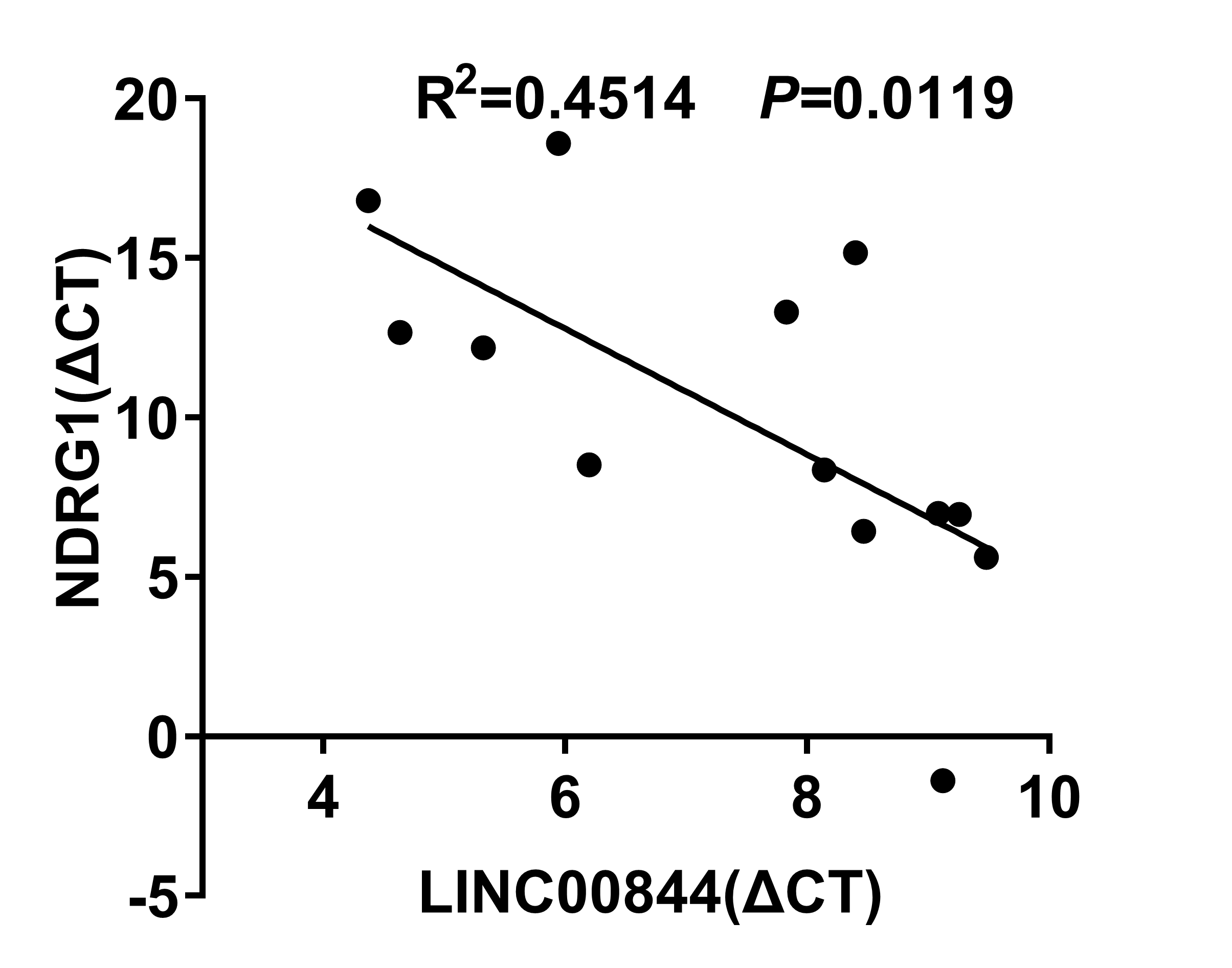

Supplement: Figure S2 [file peerj-08-8394-s002.png]

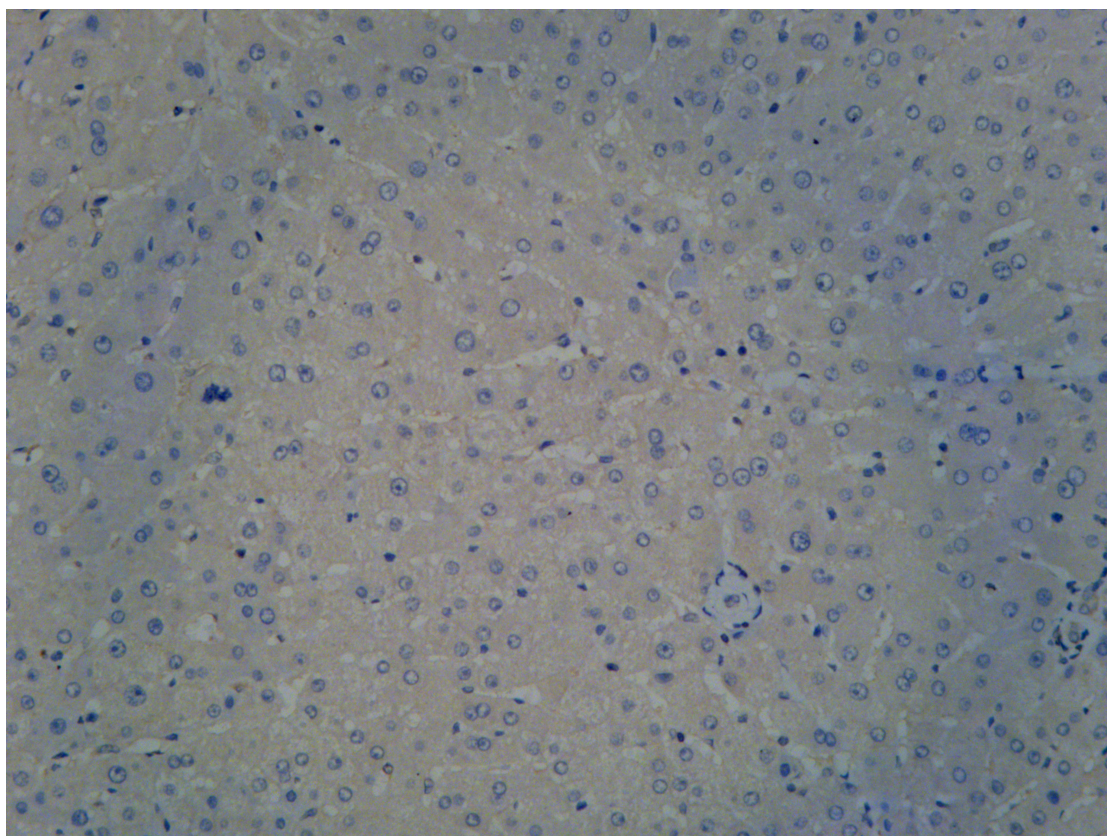

#1 Normal

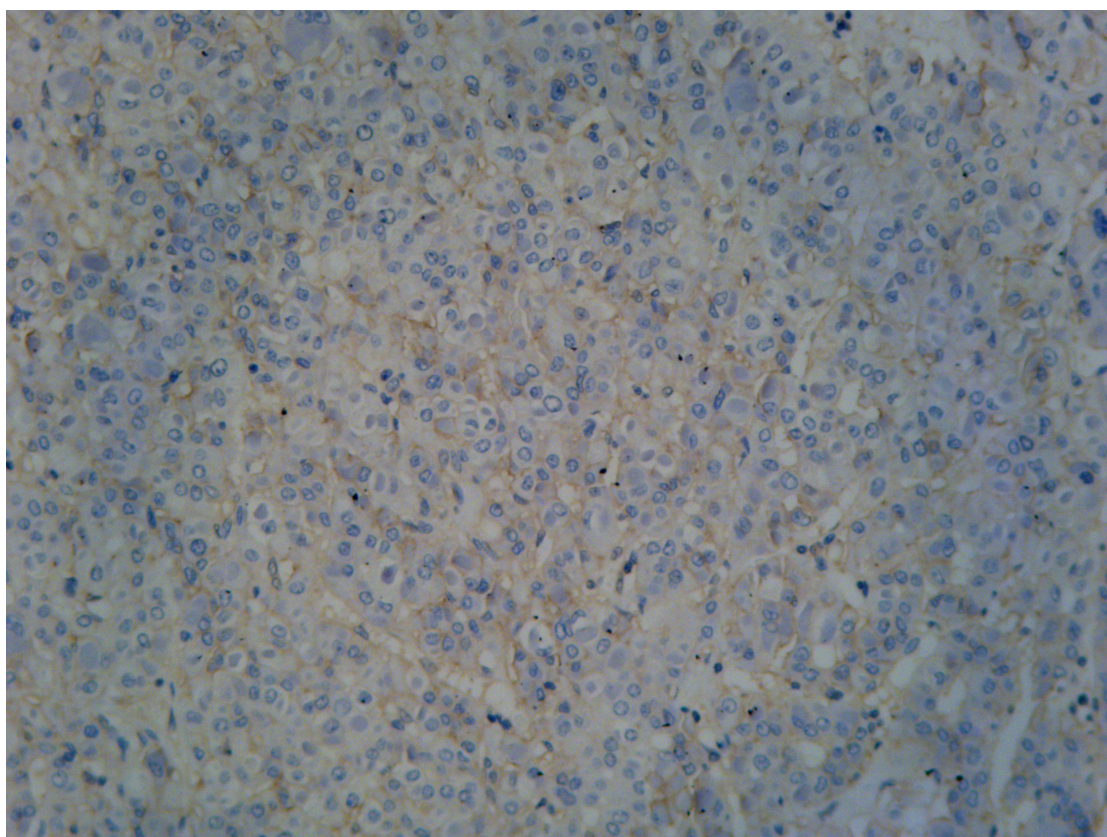

#1 Tumor

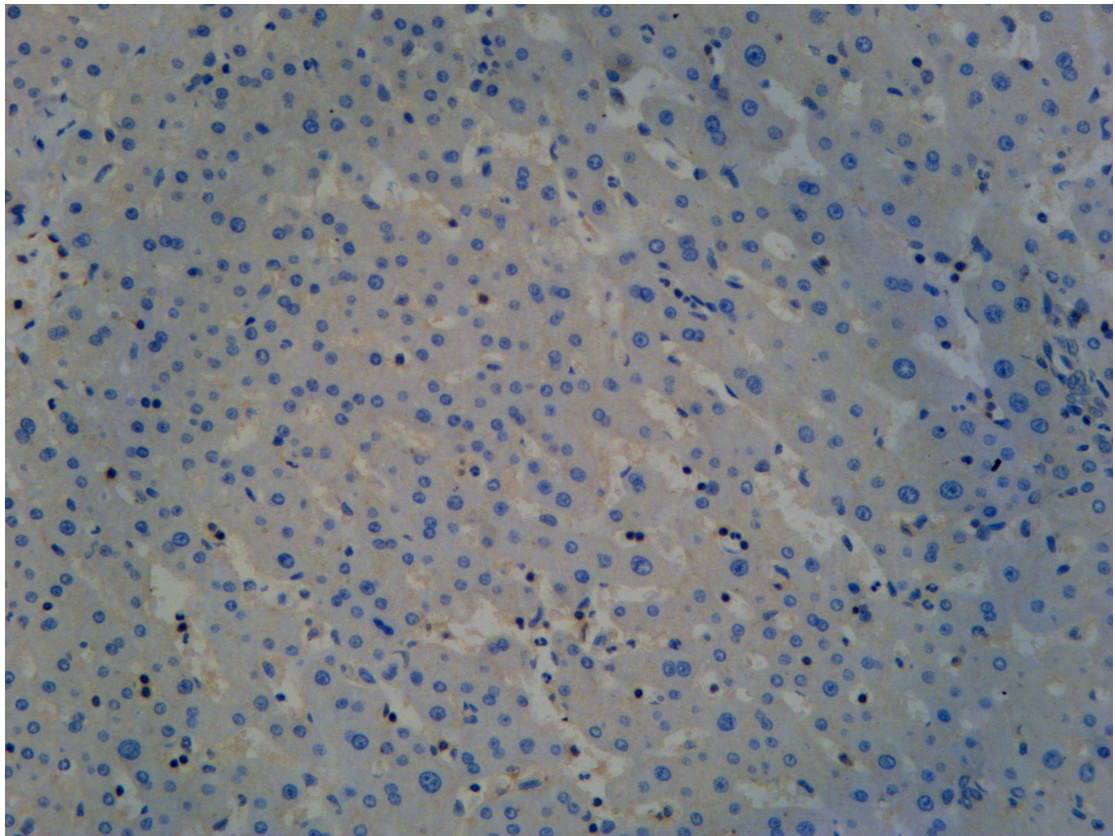

#2 Normal

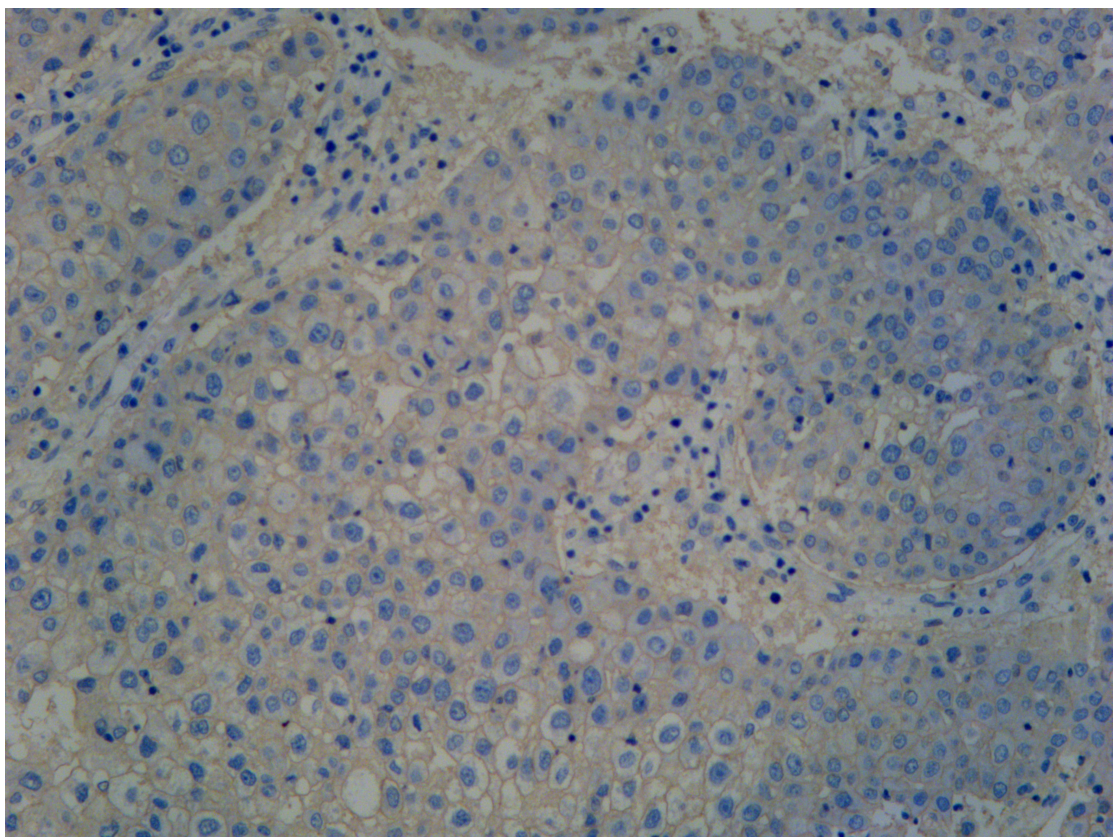

#2 Tumor

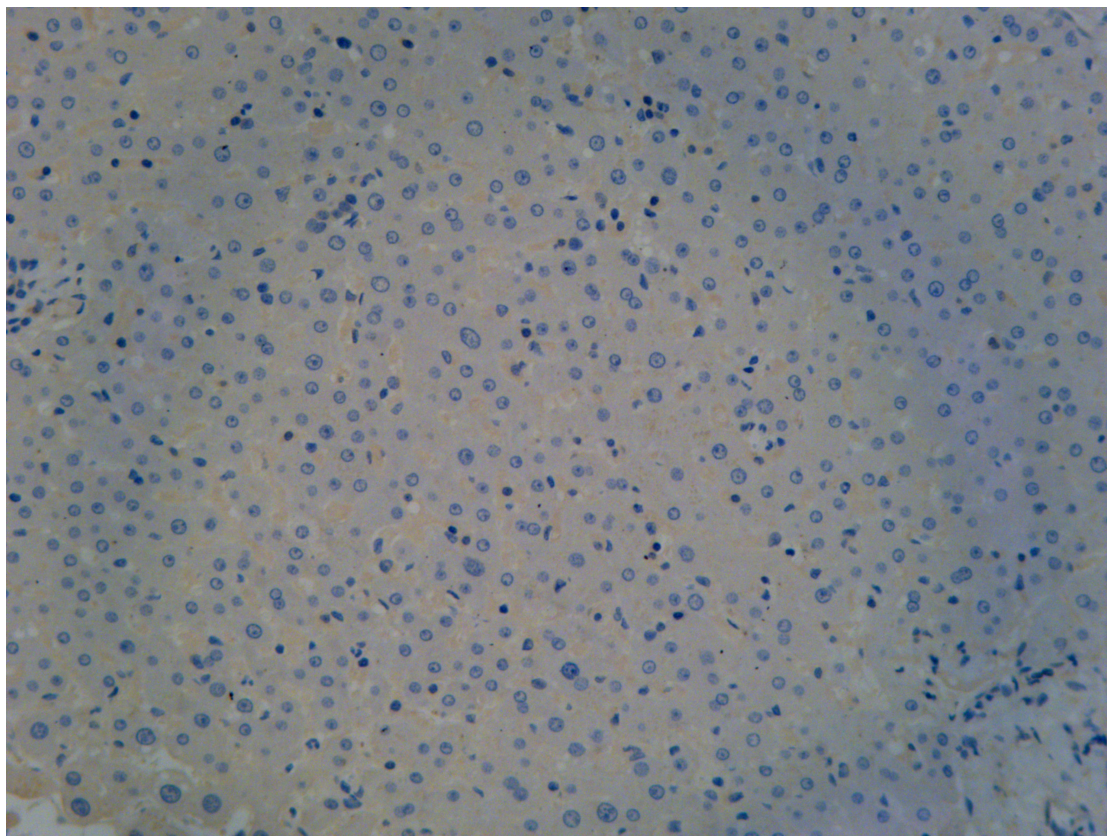

#3 Normal

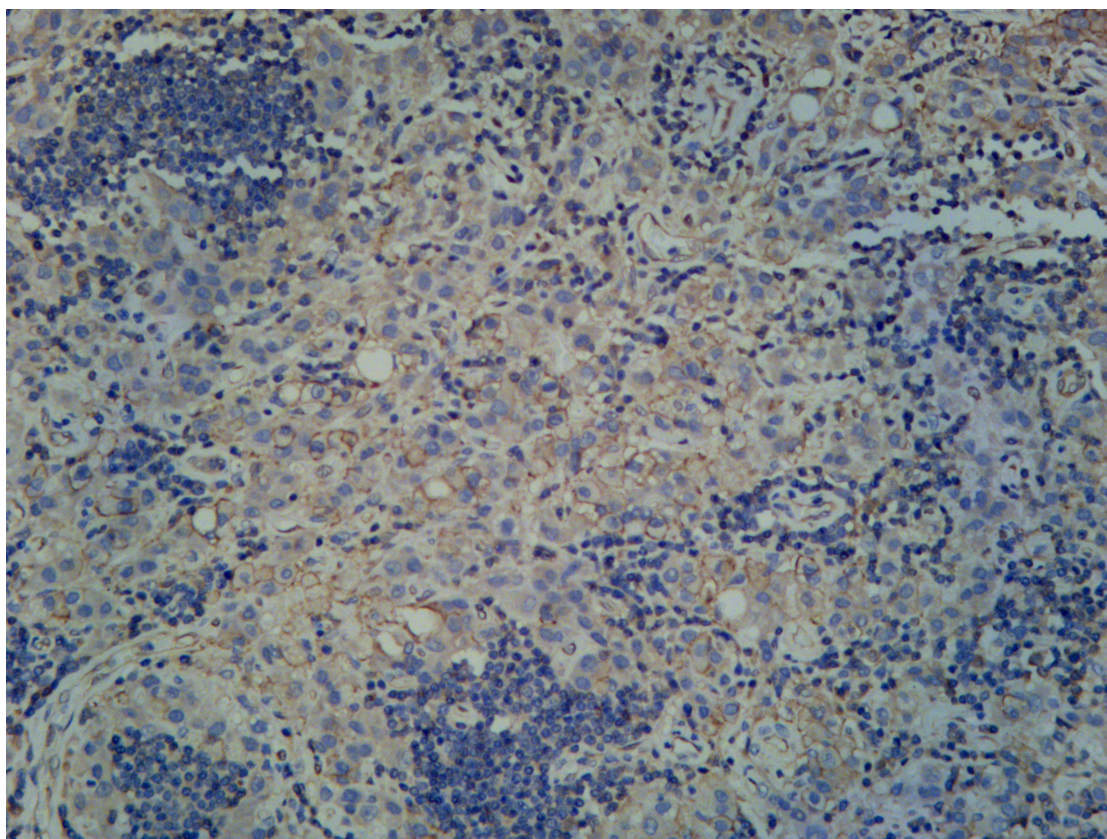

#3 Tumor

Supplement: File S13 [file peerj-08-8394-s015.pdf]

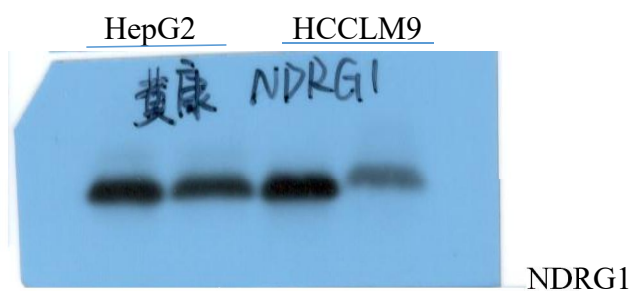

LV-NC      LV-LINC00844      LV-NC      LV-LINC00844

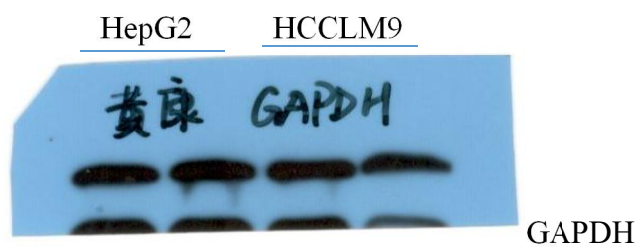

LV-NC      LV-LINC00844      LV-NC      LV-LINC00844

Supplement: File S15 [file peerj-08-8394-s017.pdf]
